# 

**Published:** 1883-01

**Authors:** 


### BOOKS AND PERIODICALS.

STUDER'S POPULAR ORNITHOLOGY — THE BIRDS OF NORTH AMERICA —We have seen many handsome books—books that challenge the admiration of persons of pronounced artistic culture—but never have we been permitted to enjoy such an exquisite volume as the one the title of which we give above.

The volume is a large quarto, bound in full Turkey morocco, and contains upwards of seven hundred illustrations, including all the birds known to exist in North America. These illustrations are works of art—truthfully drawn to nature, and faithful colorings of the beautiful creatures they are designed to illustrate.

Aside from its value as a delightful book to examine, by reason of its artistic merit, it is invaluable to the ornithologist as an aid in identifying species; is of great value to lovers of nature, who, hearing the note of a sweet songster, and, searching him out, can readily learn from this book his name and habits.

It is edited in a charming and familiar style by Jacob H. Studer, who, in producing such an elaborate and handsome volume of our birds, is entitled to the gratitude of all Americans in love with the feathered tribe. Address the publishers, Jacob H. Studer & Co., New York.

---

PROCEEDINGS OF THE AMERICAN SOCIETY OF MICROSCOPISTS, fifth annual meeting, held at Elmira, N. Y., August 15th to 18th, 1882.

This volume of proceedings does great credit to the publication committee and mainly to Prof. D. S. Kellicott, of Buffalo, whose handiwork is recognized throughout the work. It is no small undertaking to arrange so much matter as is presented in this volume of 300 pages, and have so very few errors creep in as are to be found in this one. The arrangement of and illustrations for the several papers, could not have been better. The minutes of the meeting are very full and correspondingly entertaining. Prof. Kellicott has done his work thoroughly and completely. He deserves well of the Society. The book can be had by addressing the Treasurer, Dr. George E. Fell, Buffalo, N. Y.

---

THE MEDICAL SUMMARY, a monthly medical journal, published at Linsdele, Pa., by Dr. Andrews, has been much improved in matter and appearance. It is worth many times the price asked for it.

